# Supplementary material for: Endophytic Fungi Residing within Cornus florida L. in Mid-Tennessee: Phylogenetic Diversity, Enzymatic Properties, and Potential Role in Plant Health
Source: Plants (Basel). 2024 Apr 30;13(9):1250. doi: 10.3390/plants13091250 (PMC11085766; doi:10.3390/plants13091250)
Supplement: Supplementary file 1 [file plants-13-01250-s001.zip › Supplementary Table S1 Overview of endophytic fungi.pdf]

Supplementary Table S1: Overview of endophytic fungi isolated from the stem of *C. florida* including their family, closest hit in BLAStn search with GeneBank Accesion number and percentage similarity based on ITS analysis.

ID	Identification	Family	Closest GeneBank match/Accession	Similarity bp
**A3F1**	*Xylariales* sp.	Unclassified Xyrlariales	*Xylariales* sp. (HQ608051)	492/504 (97.6%)
**A4F1**	*Dothideales* sp.	Unassigned	*Dothideales* sp. (JX188157)	520/536 (97%)
**A-A4F2**	*Hypoxylon* sp.	Hypoxylaceae	*Hypoxylon* sp. (KX722238)	518/526 (98.5%)
**A-A5F1**	*Hypoxylon perforatum*	Hypoxylaceae	*Hypoxylon perforatum* (JQ009306)	573/575 (99.6%)
**A-A5F2**	*Xylaria* sp.	Xylariaceae	*Xylaria* sp. (JQ761738)	716/717 (99.9%)
**A-A7F1**	*Seimatosporium lichenicola*	Amphisphaeriaceae	*Discostroma fuscellum* (JF320818)	550/558 (98.6%)
**A-A8F1**	*Botryosphaeria dothidea*	Botryosphaeriales	*Botryosphaeria dothidea* (KX648519)	539/539 (100%)
**A-A8F2**	*Pestalotiopsis microspora*	Sporocadaceae	*Pestalotiopsis microspora* (KM438014)	484/484 (100%)
**A-A9F1**	*Polyporales* sp.	Unassigned	*Flavodon flavus* (KY703429)	232/256 (90.6%)
**A-A9F2**	*Cytospora* sp.	Valsaceae	*Cytospora* sp. (AY188991)	550/552 (99.6%)
**A-A10F1**	*Bjerkandera adusta*	Phanerochaetaceae	*Bjerkandera adusta* (KC176334)	572/573 (99.8%)
**B-A11F1**	*Didymosphaeria variabile*	Didymosphaeriacea e	*Didymosphaeria variabile* (KX869961)	559/559 (100%)
**B-A12F1**	*Daldinia childiae*	Hypoxylaceae	*Daldinia childiae* (KU683757)	547/557 (98.2%)
**B-A12F2**	*Hypoxylon rubiginosum*	Hypoxylaceae	*Hypoxylon rubiginosum* (JQ760786)	566/577 (98.1%)

**B-A13F1**	*Nemania* sp.	Xylariaceae	*Nemania* sp. (JQ760461)	539/541 (99.6%)
**B-A15F1**	*Didymosphaeria variabile*	Didymosphaeriacea e	*Didymosphaeria variabile* (KX869961)	553/554 (99.8%)
**B-A17F1**	*Whalleya microplaca*	Xylariaceae	*Whalleya microplaca* (EF026129)	554/559 (99.1%)
**B-A17F2**	*Rosellinia corticium*	Xylariaceae	*Rosellinia corticium* (KC311485)	523/528 (99%)
**B-A19F1**	*Xylaria* sp.	Xylariaceae	*Xylaria* sp. (KU683939)	749/750 (99.9%)
**B-A20F1**	*Hypoxylon perforatum*	Hypoxylaceae	*Hypoxylon perforatum* (KY950447)	575/578 (99.5%)
**B-A20F2**	*Hypoxylon howeanum*	Hypoxylaceae	*Hypoxylon howeanum* (HE774495)	571/574 (99.5%)
**C-A21F1**	*Seimatosporium lichenicola*	Amphisphaeriaceae	*Discostroma fuscellum* (JF320818)	519/528 (98.3%)
**C-A21F2**	*Dothideales* sp.	Unassigned	*Dothideales* sp. JX188157	541/558 (96.9%)
**C-A22F1**	*Nigrospora sphaerica*	Trichosphaeriales	*Nigrospora sphaerica* (KF171529)	494/495 (99.8%)
**C-A22F2**	*Hypoxylon perforatum*	Hypoxylaceae	*Hypoxylon perforatum* (KY950447)	587/593 (99%)
**C-A22F3**	*Peniophora lycii*	Peniophoraceae	*Peniophora lycii* (JX046435)	586/592 (99%)
**C-A22F4**	Basidiomycota sp.	Unassigned	Basidiomycota sp. (GU566231)	586/593 (98.8%)
**C-A23F1**	*Daldinia childiae*	Hypoxylaceae	*Daldinia childiae* (KU683757)	512/513 (99.8%)
**C-A23F2**	*Hypoxylon fuscum*	Hypoxylaceae	*Hypoxylon fuscum* (JN979424)	553/566 (97.7%)
**C-A23F3**	*Whalleya microplaca*	Xylariaceae	*Whalleya microplaca* (EF026129)	565/571 (98.9%)
**C-A24F1**	*Pestalotiopsis* sp.	Sporocadaceae	*Pestalotiopsis* sp. (KJ542337)	621/624 (99.5%)
**C-A24F2**	*Dothideales* sp.	Unassigned	*Dothideales* sp. (JX188157)	539/559 (96.4%)

**C-A26F2**	*Hypoxylon rubiginosum*	Hypoxylaceae	*Hypoxylon rubiginosum* (MH178694)	505/511 (98.8%)
**C-A26F3**	*Hypoxylon submonticulosum*	Hypoxylaceae	*Hypoxylon submonticulosum* (MG004779)	518/519 (99.8%)
**C-A26F4**	*Hypoxylon perforatum*	Hypoxylaceae	*Hypoxylon perforatum* (KY950447)	572/575 (99.5%
**C-A26F5**	*Hypoxylon rubiginosum*	Hypoxylaceae	*Hypoxylon rubiginosum* (MH178694)	505/510 (99%)
**C-A26F8**	*Hypoxylon* sp.	Hypoxylaceae	*Hypoxylon* sp. (KX722238)	526/535 (98.3%)
**C-A27F1**	*Hypoxylon perforatum*	Hypoxylaceae	*Hypoxylon perforatum* (KY950447)	563/570 (98.8%)
**C-A27F2**	*Ascochyta medicaginicola*	Didymellaceae	*Ascochyta medicaginicola* (KX065046)	498/500 (99.6%)
**C-A27F3**	*Dothideales* sp.	Unassigned	*Dothideales* sp. (JX188157)	527/544 (96.9%)
**C-A28F1**	*Cladosporium cladosporioides*	Cladosporiaceae	*Cladosporium cladosporioides* (MF472939)	481/481 (100%)
**C-A30F1**	*Xylaria* cf. *heliscus*	Xylariaceae	*Xylaria* cf. *heliscus* (JQ761642)	514/514 (100%)
**D-A31F1**	*Hypoxylon fragiforme*	Hypoxylaceae	*Hypoxylon fragiforme* (KU684022)	556/565 (98.4%)
**D-A31F2**	*Mycosphaerella aurantia*	Mycosphaerellaceae	*Mycosphaerella aurantia* (EU851913)	487/489 (99.6%)
**D-A32F1**	*Coniozyma* sp.	Dothideaceae	*Coniozyma* sp. (KF646090)	526/539 (97.6%)
**D-A33F1**	*Daldinia childiae*	Hypoxylaceae	*Daldinia childiae* (KU683757)	520/521 (99.8%)
**D-A33F4**	*Daldinia childiae*	Hypoxylaceae	*Daldinia childiae* (KU683757)	511/512 (99.8%)
**D-A34F1**	*Diplodia seriata*	Botryosphaeriales	*Diplodia seriata* (KR822800)	542/544 (99.6%)

**D-A34F2**	*Nemania serpens*	Xylariaceae	*Nemania serpens* (KU683860)	619/623 (99.4%)
**D-A35F1**	*Hypoxylon perforatum*	Hypoxylaceae	*Hypoxylon perforatum* (JQ009308)	570/579 (98.5%)
**D-A35F2**	*Hypoxylon perforatum*	Hypoxylaceae	*Hypoxylon perforatum* (JQ009308)	513/518 (99%)
**D-A35F3**	*Hypoxylon* sp.	Hypoxylaceae	*Hypoxylon* sp. (JQ761436)	612/615 (99.5%)
**D-A36F2**	Fungal sp.	Unassigned	Uncultured fungus clone (KF800545)	382/452 (84.5%)
**D-A37F1**	*Didymosphaeria variabile*	Didymosphaeriacea e	*Didymosphaeria variabile* (KX869961)	561/561 (100%)
**D-A37F2**	*Xylaria* sp.	Xylariaceae	*Xylaria* sp. (JQ761730)	719/722 (99.6%
**D-A38F1**	*Daldinia childiae*	Hypoxylaceae	*Daldinia childiae* (KU683757)	455/470 (96.8%)
**D-A40F1**	*Annulohypoxylon annulatum*	Hypoxylaceae	*Annulohypoxylon annulatum* (NR_153579)	455/463 (98.3%)
**D-A40F2**	*Nemania serpens*	Xylariaceae	*Nemania serpens* (MG004726)	527/527 (100%)
**D-A40F3**	*Hypoxylon submonticulosum*	Hypoxylaceae	*Hypoxylon submonticulosum* (MG004779)	514/516 (99.6%)
**E-A41F1**	*Colletotrichum acutatum*	Glomerellaceae	*Colletotrichum acutatum* (KM520005)	530/534 (99.3%)
**E-A41F3**	*Hypoxylon* sp.	Hypoxylaceae	*Hypoxylon* sp. (MH178703)	512/515 (99.4%)
**E-A41F4**	*Colletotrichum gloeosporioides*	Glomerellaceae	*Colletotrichum gloeosporioides* (KX578792)	535/540 (99.1%)
**E-A42F1**	*Diaporthe* sp.	Diaporthaceae	*Diaporthe* sp. (KU375739)	536/539 (99.4%)
**E-A42F3**	*Pleosporales* sp.	Unassigned	*Camarographium koreanum* (JQ044432)	451/503 (89.7%)

**E-A42F4**	*Xylaria* cf. *heliscus*	Xylariaceae	*Xylaria* cf. *heliscus* (JQ761642)	540/548 (98.5%)
**E-A42F5**	*Stereum complicatum*	Stereaceae	*Stereum complicatum* (KU559368)	569/573 (99.3%)
**E-A43F1**	*Daldinia childiae*	Hypoxylaceae	*Daldinia childiae* (KU683757)	532/536 (99.3%)
**E-A43F2**	*Hypoxylon* sp.	Hypoxylaceae	*Hypoxylon perforatum* (KM513593)	577/605 (95.4%)
**E-A43F4**	*Diplodia seriata*	Botryosphaeriales	*Diplodia seriata* (KT004551)	535/536 (99.8%)
**E-A44F1**	*Pestalotiopsis microspora*	Sporocadaceae	*Pestalotiopsis microspore* (GU441590)	535/535 (100%)
**E-A44F2**	*Pestalotiopsis vismiae*	Sporocadaceae	*Pestalotiopsis vismiae* (KY810494)	545/545 (100%)
**E-A44F3**	*Stereum complicatum*	Stereaceae	*Stereum complicatum* (KU559368)	556/559 (99.5%)
**E-A44F4**	*Paraconiothyrium brasiliense*	Didymosphaeriacea e	*Paraconiothyrium Brasiliense* (JF439492)	553/553 (100%)
**E-A45F1**	*Didymosphaeria variabile*	Didymosphaeriacea e	*Didymosphaeria variabile* (KX8699610	555/558 (99.5%)
**E-A46F1**	*Diaporthe* cf. *nobilis*	Diaporthaceae	*Diaporthe* cf. *nobilis* (KC3431480	464/466 (99.6%)
**E-A46F2**	*Xylaria* sp.	Xylariaceae	*Xylaria* sp. (AB449101)	486/505 (96.2%)
**E-A46F3**	*Pestalotiopsis microspora*	Sporocadaceae	*Pestalotiopsis microspore* (KM041703)	500/501 (99.8%)
**E-A47F1**	*Peniophora* cf. *limitata*	Peniophoraceae	*Peniophora* cf. *limitata* (KP814438)	561/568 (98.8%)
**E-A47F2**	*Hypoxylon perforatum*	Hypoxylaceae	*Hypoxylon perforatum* (KJ957773)	523/530 (98.7%)
**E-A47F4**	*Xylomelasma* sp.	Unassigned	*Xylomelasma* sp. (FR837913)_	552/556 (99.3%)

**E-A47F5**	*Hypoxylon fragiforme*	Hypoxylaceae	*Hypoxylon fragiforme* (KU684022)	576/576 (100%)
**E-A47F6**	*Seimatosporium lichenicola*	Amphisphaeriaceae	*Discostroma fuscellum* (JF320818)	545/552 (98.7%)
**E-A48F1**	*Daldinia childiae*	Hypoxylaceae	*Daldinia childiae* (KU683757)	521/522 (99.8%)
**E-A48F2**	*Seimatosporium lichenicola*	Amphisphaeriaceae	*Discostroma fuscellum* (JF320818)	538/540 (99.6%)
**E-A48F3**	*Seimatosporium lichenicola*	Amphisphaeriaceae	*Discostroma fuscellum* (JF320818)	547/553 (98.9%)
**E-A48F4**	*Daldinia childiae*	Hypoxylaceae	*Daldinia childiae* (KU683757)	521/522 (99.8%)
**E-A48F6**	*Phyllosticta pyrolae*	Phyllostictaceae	*Phyllosticta pyrolae* (AF312010)	583/591 (98.6%)
**E-A48F7**	*Hypoxylon perforatum*	Hypoxylaceae	*Hypoxylon perforatum* (JQ009306)	552/562 (98.2%)
**E-A48F9**	*Whalleya microplaca*	Xylariaceae	*Whalleya microplaca* (EF026129)	548/550 (99.6%)
**E-A48F10**	*Daldinia childiae*	Hypoxylaceae	*Daldinia childiae* (KU683757)	521/522 (99.8%)
**E-A48F12**	*Alternaria alternata*	Pleosporaceae	*Alternaria alternate* (KX064976)	510/510 (100%)
**E-A48F13**	*Seimatosporium lichenicola*	Amphisphaeriaceae	*Seimatosporium lichenicola* (MF069255)	528/530 (99.6%)
**E-A49F1**	*Whalleya microplaca*	Xylariaceae	*Whalleya microplaca* (EF026129)	512/519 (98.7%)
**E-A49F2**	*Hypoxylon perforatum*	Hypoxylaceae	*Hypoxylon perforatum* (KY950447)	575/580 (99.1%)
**E-A50F1**	*Diplodia seriata*	Botryosphaeriaceae	*Diplodia seriata* (KR822800)	516/518 (99.6%)
**F-A51F1**	*Didymella* sp.	Didymellaceae	*Didymella* sp. (MF065737)	488/491 (99.4%)
**F-A51F2**	*Pestalotiopsis hainanensis*	Sporocadaceae	*Pestalotiopsis hainanensis*	540/540 (100%)

			(KF551573)	
**F-A51F3**	*Daldinia childiae*	Hypoxylaceae	*Daldinia childiae* KU683757	522/531 (98.3%)
**F-A51F4**	*Didymella* sp.	Didymellaceae	*Didymella* sp. (MF065737)	495/495 (100%)
**F-A51F5**	*Nothophoma quercina*	Didymellaceae	*Nothophoma quercina* (LT592929)	490/490 (100%)
**F-A52F1**	*Paraconiothyrium brasiliense*	Didymosphaeriacea e	*Paraconiothyrium Brasiliense* (JF439492)	552/552 (100%)
**F-A52F2**	Fungal sp.	Unassigned	Uncultured fungus clone (MF976687)	465/519 (89.6%)
**F-A53F1**	*Nigrospora sphaerica*	Trichosphaeriaceae	*Nigrospora sphaerica* (KF171529)	501/501 (100%)
**F-A53F2**	*Hypoxylon* sp.	Hypoxylaceae	*Hypoxylon* sp. (JQ761682)	611/617 (99%)
**F-A54F1**	*Xylaria* sp.	Xylariaceae	*Xylaria arbuscular* (MG004732)	482/500 (96.4%)
**F-A54F2**	*Pleosporales* sp.	Unclassified Pleosporales	*Pleosporales* sp. (KX148623)	429/440 (97.5%)
**F-A54F3**	*Valsa sordida*	Valsaceae	*Cytospora chrysosperma* (KJ739458)	561/570 (98.4%)
**F-A54F4**	*Didymella* sp.	Didymellaceae	*Didymella* sp. (MF065737)	491/492 (99.8%)
**F-A54F5**	*Hypoxylon perforatum*	Hypoxylaceae	*Hypoxylon perforatum* (FJ464593)	575/580 (99.1%)
**F-A55F1**	*Coniothyrium* sp.	Coniothyriaceae	*Coniothyrium* sp. (KY047484)	488/488 (100%)
**F-A55F2**	*Nothophoma quercina*	Didymellaceae	*Nothophoma quercina* (LT592929)	488/488 (100%)
**F-A61F1**	*Didymella glomerata*	Didymellaceae	*Didymella glomerate* (KX064999)	490/490 (100%)
**F-A61F2**	*Seimatosporium lichenicola*	Amphisphaeriaceae	*Seimatosporium lichenicola* (KT000158)	514/518 (99.2%)

**F-A61F3**	*Ascochyta medicaginicola*	Didymellaceae	*Phoma medicaginis* (KT192351)	490/490 (100%)
**F-A61F4**	*Pestalotiopsis microspora*	Sporocadaceae	*Pestalotiopsis microspore* (KM438014)	498/498 (100%)
**F-A62F1**	*Didymosphaeria variabile*	Didymosphaeriacea e	*Didymosphaeria variabile* (KX869961)	555/555 (100%)
**F-A63F1**	*Didymella glomerata*	Didymellaceae	*Didymella glomerata* (KX064999)	490/491 (99.8%)
**F-A63F2**	*Didymella glomerata*	Didymellaceae	*Didymella glomerata* (KX064999)	492/492 (100%)
**F-A63F3**	*Ascochyta medicaginicola*	Didymellaceae	*Ascochyta medicaginicola* (KX064990)	491/491 (100%)
**F-A64F1**	*Epicoccum nigrum*	Didymellaceae	*Epicoccum nigrum* (EU821486)	491/491 (100%)
**F-A64F3**	*Didymella* sp.	Didymellaceae	*Didymella* sp. (MF065737)	496/496 (100%)
**F-A64F5**	*Coniothyrium* sp.	Coniothyriaceae	*Coniothyrium* sp. (KY047484)	490/490 (100%)
**F-A64F6**	*Cytospora* sp.	Valsaceae	*Cytospora* sp. (KC342485)	549/557 (98.6%)
**G-A66F1**	*Didymosphaeria variabile*	Didymosphaeriacea e	*Didymosphaeria variabile* (KX869961)	547/548 (99.8%)
**G-A66F2**	*Pestalotiopsis* sp.	Sporocadaceae	*Pestalotiopsis* sp. (KX343175)	541/542 (99.8%)
**G-A66F3**	*Ascochyta medicaginicola*	Didymellaceae	*Ascochyta medicaginicola* (KX064990)	500/500 (100%)
**G-A67F2**	*Didymella glomerata*	Didymellaceae	*Didymella glomerate* (MG372318)	491/491 (100%)
**G-A67F3**	*Didymella glomerata*	Didymellaceae	*Peyronellaea glomerate* (KT192211)	480/482 (99.6%)
**G-A67F4**	*Pleosporales* sp.	unclassified Pleosporales	*Pleosporales* sp. (KR909157)	510/516 (98.8%)
**G-A68F1**	*Ascochyta medicaginicola*	Didymellaceae	*Ascochyta medicaginicola* (KX065046)	490/490 (100%)

**G-A68F2**	*Pestalotiopsis mangiferae*	Sporocadaceae	*Pestalotiopsis mangiferae* (JX305692)	478/478 (100%)
**G-A68F3**	*Ceuthospora pinastri*	Phacidiaceae	*Ceuthospora pinastri* (FR717225)	486/494 (98.4%)
**G-A68F4**	*Pestalotiopsis* sp.	Sporocadaceae	*Pestalotiopsis* sp. (JN418796)	492/494 (99.6%)
**G-A69F1**	*Dothideales* sp.	Unassigned	*Dothideales* sp. (JX188157)	520/537 (96.8%)
**G-A70F1**	*Coniothyrium* sp.	Coniothyriaceae	*Coniothyrium* sp. (KY047484)	489/489 (100%)
**G-A70F2**	*Nothophoma quercina*	Didymellaceae	*Nothophoma quercina* (LT592929)	488/488 (100%)
**G-A71F1**	*Ascochyta medicaginicola*	Didymellaceae	*Ascochyta medicaginicola* (KX065046)	496/496 (100%)
**G-A71F2**	*Phoma aliena*	Didymellaceae	*Phoma aliena* (KX869951)	500/500 (100%)
**G-A71F3**	*Nigrospora sphaerica*	Trichosphaeriaceae	*Nigrospora sphaerica* (KF171529)	502/503 (99.8%)
**G-A72F1**	*Nothophoma quercina*	Didymellaceae	*Nothophoma quercina* (LT592929)	482/485 (99.4%)
**G-A72F2**	*Didymella* sp.	Didymellaceae	*Didymella* sp. (MF065737)	477/490 (97.4%)
**G-A72F3**	*Nothophoma quercina*	Didymellaceae	*Nothophoma quercina* (KU973713)	497/499 (99.6%)
**H-A56F1**	*Pestalotiopsis microspora*	Sporocadaceae	*Pestalotiopsis microspore* (KM041703)	491/495 (99.2%)
**H-A56F4**	*Phoma aliena*	Didymellaceae	*Phoma aliena* (KX869951)	500/500 (100%)
**H-A56F5**	*Diplodia seriata*	Botryosphaeriaceae	*Diplodia seriata* (KY385656)	535/536 (99.8%)
**H-A56F6**	*Ascochyta medicaginicola*	Didymellaceae	*Ascochyta medicaginicola* (KX064990)	503/505 (99.6%)
**H-A56F7**	*Pestalotiopsis vismiae*	Sporocadaceae	*Pestalotiopsis vismiae* (KM513583)	559/562 (99.5%)

**H-A57F1**	*Cytospora* sp.	Valsaceae	*Cytospora sophorae* (KC880147)	532/549 (96.9%)
**H-A57F2**	*Cytospora* sp.	Valsaceae	*Cytospora sophorae* (KC880147)	548/559 (98%)
**H-A57F3**	*Didymosphaeria variabile*	Didymosphaeriaceae	*Didymosphaeria variabile* (KX869961)	556/556 (100%)
**H-A57F6**	*Seiridium* sp.	Sporocadaceae	*Seiridium* sp. (JN198507)	521/529 (98.5%)
**H-A57F7**	*Pestalotiopsis vismiae*	Sporocadaceae	*Pestalotiopsis vismiae* (KM513583)	541/541 (100%)
**H-A57F8**	*Didymosphaeria variabile*	Didymosphaeriaceae	*Didymosphaeria variabile* (KX869961)	567/569 (99.6%)
**H-A58F1**	*Didymosphaeria variabile*	Didymosphaeriaceae	*Didymosphaeria variabile* (KX869961)	563/563 (100%)
**H-A58F2**	*Hypoxylon perforatum*	Hypoxylaceae	*Hypoxylon perforatum* (JQ009306)	564/565 (99.8%)
**H-A58F3**	*Hypoxylon perforatum*	Hypoxylaceae	*Hypoxylon perforatum* (JQ009306)	574/578 (99.3%)
**H-A59F1**	*Paraconiothyrium brasiliense*	Didymosphaeriaceae	*Paraconiothyrium Brasiliense* (JF439492)	562/562 (100%)
**H-A59F2**	*Didymella glomerata*	Didymellaceae	*Didymella glomerate* (KX064999)	490/490 (100%)
**H-A59F3**	*Phoma* sp.	Didymellaceae	*Phoma* sp. (FJ949079)	508/509 (99.8%)
**H-A59F4**	*Ceuthospora pinastri*	Phacidiaceae	Ceuthospora pinastri (FR717225)	504/509 (99%)
**H-A60F2**	*Pestalotiopsis* sp.	Sporocadaceae	*Pestalotiopsis* sp. (KP217176)	508/509 (99.8%)
**H-A60F3**	*Microdiplodia* sp.	Botryosphaeriaceae	*Microdiplodia* sp. (KM877481)	547/548 (99.8%)
