# Supplementary material for: Endophytic Fungi Residing within Cornus florida L. in Mid-Tennessee: Phylogenetic Diversity, Enzymatic Properties, and Potential Role in Plant Health
Source: Plants (Basel). 2024 Apr 30;13(9):1250. doi: 10.3390/plants13091250 (PMC11085766; doi:10.3390/plants13091250)
Supplement: Supplementary file 1 [file plants-13-01250-s001.zip › Supplementary Table S2 Relative frequency distribution.pdf]

Supplementary Table S2: Relative frequency distribution of endophytic fungal communities isolated from *C. florida* stem collected from eight different locations in middle Tennessee.

	**Relative frequency (%) at A-H locations[z]**								**Mea n**
	**A**	**B**	**C**	**D**	**E**	**F**	**G**	**H**	
**Ascomycota (96.5)**									
**Sordariomycetes (49.3)**									
**Xylariales**	71.1	71.1	50.9	59.4	43.9	27.7	10.0	23.1	**43.9**
Annulohypoxylon annulatum	-	-	-	18.8	-	-	-	-	**1.6**
Daldinia childiae	-	11.1	5.7	12.5	8.8	-	-	-	**4.6**
***Hypoxylon* sp.**	5.3	-	1.9	3.1	5.3	1.5	-	-	**2.2**
Hypoxylon fragiforme	-	-	-	3.1	1.8	-	-	-	**0.54**
Hypoxylon fuscum	-	-	1.9	-	-	-	-	-	**0.27**
Hypoxylon howeanum	-	11.1	-	-	-	-	-	-	**1.4**
Hypoxylon perforatum	10.5	2.2	11.3	6.3	5.3	3.1	-	5.1	**5.4**
Hypoxylon rubiginosum	-	2.2	3.8	-	-	-	-	-	**0.81**
Hypoxylon submonticulosum	-	-	1.9	3.1	-	-	-	-	**0.54**
***Nemania* sp.**	-	6.7	-	-	-	-	-	-	**0.81**
Nemania serpens	-	-	-	9.4	-	-	-	-	**0.81**
***Pestalotiopsis* sp.**	-	-	15.1	-	-	-	5.0	2.6	**3.0**
Pestalotiopsis hainanensis	-	-	-	-	-	6.2	-	-	**1.1**
Pestalotiopsis mangiferae	-	-	-	-	-	-	5.0	-	**0.54**
Pestalotiopsis microspora	23.7	-	-	-	5.3	7.7	-	5.1	**5.2**
Pestalotiopsis vismiae	-	-	-	-	1.8	-	-	7.7	**1.1**
Rosellinia corticium	-	2.2	-	-	-	-	-	-	**0.27**
Seimatosporium lichenicola	7.9	-	3.8	-	8.8	1.5	-	-	**3.0**
***Seiridium* sp.**	-	-	-	-	-	-	-	2.6	**0.27**
Whalleya microplaca	-	20.0	1.9	-	3.5	-	-	-	**3.3**
***Xylaria* sp.**	5.3	15.6	-	3.1	1.8	7.7	-	-	**4.4**
Xylaria* cf. *heliscus	-	-	5.7	-	1.8	-	-	-	**1.1**

**Unclassified Xyrlariales**	18.4	-	-	-	-	-	-	-	**1.9**
**Diaporthales**	5.3	-	-	-	5.3	3.1	-	5.1	**2.43**
Cytospora **sp.**	5.3	-	-	-	-	1.5	-	5.1	**1.4**
Diaporthe **cf.** *nobilis*	-	-	-	-	3.5	-	-	-	**0.54**
Diaporthe **sp.**	-	-	-	-	1.8	-	-	-	**0.27**
Valsa sordida	-	-	-	-	-	1.5	-	-	**0.27**
**Glomerellales**	-	-	-	-	3.5	-	-	-	**0.54**
Colletotrichum acutatum	-	-	-	-	1.8	-	-	-	**0.27**
Colletotrichum gloeosporioides	-	-	-	-	1.8	-	-	-	**0.27**
**Trichosphaeriales**	-	-	5.7	-	-	4.6	2.5	-	**1.9**
Nigrospora sphaerica	-	-	5.7	-	-	4.6	2.5	-	**1.9**
**Xylomelasma**									
**Xylomelasma sp.**	-	-	-	-	1.8	-	-	-	**0.27**
**Dothideomycetes (45.5)**									
**Dothideales**	2.6	-	20.7	9.4	-	-	2.5	-	**4.4**
Coniozyma **sp.**	-	-	-	9.4	-	-	-	-	**0.81**
Dothideales **sp.**	2.6	-	20.7	-	-	-	2.5	-	**4.4**
**Botryosphaeriales**	10.5	-	-	18.8	7.0	-	-	5.1	**4.3**
Botryosphaeria dothidea	10.5	-	-	-	-	-	-	-	**1.1**
Diplodia seriata	-	-	-	18.8	5.3	-	-	5.1	**3.0**
Phyllosticta pyrolae	-	-	-	-	1.8	-	-	-	**0.27**
**Pleosporales**	-	28.9	1.9	3.1	31.6	63.1	82.5	56.4	**34.9**
Alternaria alternata	-	-	-	-	1.8	-	-	-	**0.27**
Ascochyta medicaginicola	-	-	1.9	-	-	3.1	10.0	5.1	**2.7**
Coniothyrium **sp.**	-	-	-	-	-	10.8	7.5	-	**2.4**
Didymella **sp.**	-	-	-	-	-	15.4	2.5	-	**3.0**
Didymella glomerata	-	-	-	-	-	15.4	30.0	10.3	**7.1**
Didymosphaeria variabile	-	28.9	-	3.1	26.3	3.1	12.5	12.8	**11.1**
Epicoccum nigrum	-	-	-	-	-	4.6	-	-	**0.81**
Nothophoma quercina	-	-	-	-	-	7.7	5.0	-	**2.2**
Paraconiothyrium brasiliense	-	-	-	-	1.8	1.5	-	15.4	**2.2**

***Pleosporales* sp.**	-	-	-	-	-	1.5	7.5	-	**1.4**
***Phoma* sp.**	-	-	-	-	-	-	-	10.3	**1.1**
Phoma aliena	-	-	-	-	-	-	2.5	2.6	**0.54**
**Capnodiales**	-	-	13.2	3.1	-	-	-	-	**2.2**
Cladosporium cladosporioides	-	-	13.2	-	-	-	-	-	**1.9**
Mycosphaerella aurantia	-	-	-	3.1	-	-	-	-	**0.27**
**Leotiomycetes (1.4)**									
**Phacidiales**	-	-	-	-	-	-	2.5	10.3	**1.4**
Ceuthospora pinastri	-	-	-	-	-		2.5	10.3	**1.4**
**Basidiomycota (3.0)**									
**Agaricomycetes (2.4)**									
**Polyporales**	10.5	-	-	-	-	-	-	-	**1.1**
Bjerkandera adusta	7.9	-	-	-	-	-	-	-	**0.81**
***Polyporales* sp.**	2.6	-	-	-	-	-	-	-	**0.27**
**Russulales**	-	-	1.9	-	7.0	-	-	-	**1.4**
Peniophora* cf. *limitata	-	-	-	-	3.5	-	-	-	**0.54**
Peniophora lycii	-	-	1.9	-	-	-	-	-	**0.27**
Stereum complicatum	-	-	-	-	3.5	-	-	-	**0.54**
**Basidiomycetes sp.**	-	-	3.8	-	-	-	-	-	**0.54**
**Unknown (0.81)**									
**Fungal sp. A36F2 (0.54)**									
**Fungal sp. A52F2 (0.27)**									

[z] Locations A-H, in which location A-E are in Warren County (McMinnville), F and G in Davidson County (Nashville) and H is a Ruterford county (Mufreesboro) location.
