# Supplementary material for: Endophytic Fungi Residing within Cornus florida L. in Mid-Tennessee: Phylogenetic Diversity, Enzymatic Properties, and Potential Role in Plant Health
Source: Plants (Basel). 2024 Apr 30;13(9):1250. doi: 10.3390/plants13091250 (PMC11085766; doi:10.3390/plants13091250)
Supplement: Supplementary file 1 [file plants-13-01250-s001.zip › Supplementary Table S3 Pathogenicity test.pdf]

Supplementary Table S3: Pathogenicity test of endophytic fungi isolated from healthy *Cornus florida*.

Endophytic fungi	Code	Pathogenic/Non-pathogenic
***Hypoxylon* sp.**	**A-A4F2**	Non-pathogenic
Hypoxylon perforatum	**A-A5F1**	Non-pathogenic
Seimatosporium lichenicola	**A-A7F1**	Non-pathogenic
Botryosphaeria dothidea	**A-A8F1**	Pathogenic
Pestalotiopsis microspora	**A-A8F2**	Pathogenic
***Polyporales* sp.**	**A-A9F1**	Non-pathogenic
***Cytospora* sp.**	**A-A9F2**	Non-pathogenic
Bjerkandera adusta	**A-A10F1**	Non-pathogenic
Didymosphaeria variabile	**B-A11F1**	Pathogenic
Daldinia childiae	**B-A12F1**	Non-pathogenic
Hypoxylon rubiginosum	**B-A12F2**	Non-pathogenic
***Nemania* sp.**	**B-A13F1**	Non-pathogenic
Didymosphaeria variabile	**B-A15F1**	Non-pathogenic
Whalleya microplaca	**B-A17F1**	Non-pathogenic
Rosellinia corticium	**B-A17F2**	Pathogenic
***Xylaria* sp.**	**B-A19F1**	Pathogenic
Hypoxylon perforatum	**B-A20F1**	Pathogenic
Hypoxylon howeanum	**B-A20F2**	Non-pathogenic
Seimatosporium lichenicola	**C-A21F1**	Non-pathogenic
***Dothideales* sp.**	**C-A21F2**	Non-pathogenic
Nigrospora sphaerica	**C-A22F1**	Non-pathogenic
Hypoxylon perforatum	**C-A22F2**	Non-pathogenic
Peniophora lycii	**C-A22F3**	Non-pathogenic
Hypoxylon fuscum	**C-A23F2**	Non-pathogenic
***Pestalotiopsis* sp.**	**C-A24F1**	Non-pathogenic
Hypoxylon submonticulosum	**C-A26F3**	Non-pathogenic
Hypoxylon perforatum	**C-A26F4**	Non-pathogenic

Hypoxylon rubiginosum	**C-A26F5**	Non-pathogenic
***Hypoxylon* sp.**	**C-A26F8**	Non-pathogenic
Hypoxylon perforatum	**C-A27F1**	Non-pathogenic
Ascochyta medicaginicola	**C-A27F2**	Non-pathogenic
Cladosporium cladosporioides	**C-A28F1**	Non-pathogenic
Xylaria* cf. *heliscus	**C-A30F1**	Non-pathogenic
Mycosphaerella aurantia	**D-A31F2**	Non-pathogenic
***Coniozyma* sp.**	**D-A32F1**	Non-pathogenic
Daldinia childiae	**D-A33F1**	Non-pathogenic
Daldinia childiae	**D-A33F4**	Non-pathogenic
Diplodia seriata	**D-A34F1**	Non-pathogenic
Hypoxylon perforatum	**D-A35F1**	Non-pathogenic
Hypoxylon perforatum	**D-A35F2**	Non-pathogenic
***Hypoxylon* sp.**	**D-A35F3**	Non-pathogenic
Didymosphaeria variabile	**D-A37F1**	Non-pathogenic
***Xylaria* sp.**	**D-A37F2**	Non-pathogenic
Daldinia childiae	**D-A38F1**	Non-pathogenic
Annulohypoxylon annulatum	**D-A40F1**	Non-pathogenic
Nemania serpens	**D-A40F2**	Pathogenic
Hypoxylon submonticulosum	**D-A40F3**	Non-pathogenic
Colletotrichum acutatum	**E-A41F1**	Pathogenic
***Hypoxylon* sp.**	**E-A41F3**	Non-pathogenic
Colletotrichum gloeosporioides	**E-A41F4**	Pathogenic
Diplodia seriata	**E-A43F4**	Pathogenic
Pestalotiopsis microspora	**E-A44F1**	Pathogenic
Stereum complicatum	**E-A44F3**	Non-pathogenic
Phyllosticta pyrolae	**E-A48F6**	Non-pathogenic
Whalleya microplaca	**E-A48F9**	Non-pathogenic
Alternaria alternata	**E-A48F12**	Pathogenic
Diplodia seriata	**E-A50F1**	Pathogenic
Daldinia childiae	**F-A51F3**	Pathogenic

Didymella **sp.**	**F-A54F4**	Non-pathogenic
Coniothyrium **sp.**	**F-A55F1**	Non-pathogenic
Phoma aliena	**H-A56F4**	Pathogenic
Cytospora sp.	**H-A57F2**	Non-pathogenic
Didymella glomerata	**F-A61F1**	Pathogenic
Ascochyta medicaginicola	**F-A61F3**	Non-pathogenic
Epicoccum nigrum	**F-A64F1**	Non-pathogenic
