# Supplementary material for: Endophytic Fungi Residing within Cornus florida L. in Mid-Tennessee: Phylogenetic Diversity, Enzymatic Properties, and Potential Role in Plant Health
Source: Plants (Basel). 2024 Apr 30;13(9):1250. doi: 10.3390/plants13091250 (PMC11085766; doi:10.3390/plants13091250)
Supplement: Supplementary file 1 [file plants-13-01250-s001.zip › Supplementary Table S4 Enzymatic activities.pdf]

Supplementary Table S4: Summarized enzymatic index values for 50 endophytic fungi screened for six extracellular enzymes.

Codes	Enzymatic index					
	Cellulase	Amylase	Laccase	Pectinase	Chitinase	Protease
**A-A4F2**	0.14±0.04	0.5±0.04	0	0	0.6±0.09	0.46±0.05
**A-A5F1**	0.45±0.01	0.54±0.1	1.0±0.2	0	0.14±0.02	0.13±0.03
**A-A7F1**	4.13±0.25	1.1±0.18	3.0±0.2	0	0	0.48±0.04
**A-A8F1**	0.07±0.01	0.08±0.0	0	0	0	0.03±0.01
**A-A8F2**	0.32±0.05	0.19±0.04	0*	1.34±0.01	0	0.42±0.03
**B-A11F1**	0.86±0.03	0	0*	0.04±0.0	0.02±0.0	1.14±0.06
**B-A13F1**	0.22±0.03	0.2±0.15	0.3±0.05	0.06±0.03	0.04±0.0	2.8±0.6
**B-A17F1**	0.15±0.0	0.02±0.0	0	0	0.06±0.0	0
**B-A17F2**	1.4±0.25	0.55±0.3	1.1±0.16	0.55±0.12	0.2±0.01	3.7±0.06
**B-A19F1**	0.15±0.01	0	0	0	0.11±0.0	0.17±0.03
**B-A20F1**	0.9±0.22	2.2±0.4	0.02±0.04	0	0.16±0.02	3.4±0.06
**C-A21F1**	5.15±0.04	0	2.4±0.2	1.43±0.18	0	0.9±0.08
**C-A22F1**	0.06±0.01	0	0*	0.08±0.02	0	1.21±0.07
**C-A22F4**	2.18±0.11	4.0±0.34	0*	0	0	0.31±0.04
**C-A23F2**	1.35±0.03	1.6±0.3	0	0	0*	0.53±0.01
**C-A24F2**	3.74±0.19	1.14±0.16	0.7±0.03	0	0	1.1±0.07
**C-A28F1**	2.48±0.28	0	0	0.76±0.18	0	0.23±0.03
**C-A29F1**	1.4±0.15	0.8±0.25	0	0	0	0.15±0.0
**D-A33F1**	0.37±0.01	0.27±0.04	0	0	0*	0.05±0.013

**D-A33F4**	0.09±0.013	0	0	0	0*	0.01±0.0
**D-A34F2**	0.02±0.003	0.5±0.06	0*	0.01±0.0	0*	2.57±0.7
**D-A40F2**	0	0	0	0	0.03±0.0	0.05±0.01
**E-A41F1**	0.03±0.0	0.33±0.02	0.02±0.02	0.3±0.06	0	2.21±0.34
**E-A41F4**	0	0	0*	0*	0*	0.1±0.02
**E-A42F1**	0.24±0.07	0	0.05±0.0	0.04±0.0	0	0.11±0.02
**E-A42F4**	0.04±0.024	0	0.15±0.02	0*	0*	0.1±0.1
**E-A42F5**	0.4±0.02	0.14±0.03	0.24±0.04	0*	0	0.14±0.02
**E-A43F3**	0.04±0.01	0	0	0	0*	0.16±0.015
**E-A43F4**	0	0	0	0	0	0*
**E-A44F1**	0.26±0.05	0.12±0.02	0.04±0.015	0	0.02±0.0	0.16±0.04
**E-A44F4**	0	0	0*	0.23±0.02	0	0.11±0.02
**E-A46F1**	0	0	0*	0.02±0.0	0.1±0.002	0.11±0.04
**E-A46F2**	0	0	0*	0.02±0.01	0*	0.06±0.006
**E-A46F3**	0.1±0.09	0	0.06±0.03	0	0	1.2±0.9
**E-A47F1**	0	0	0*	0	0*	0.31±0.02
**E-A47F4**	0.57±0.02	1.05±0.3	0.6±0.24	0	0*	0.07±0.02
**E-A47F6**	5.4±0.2	1.5±0.04	3.5±0.04	0	0	0.9±0.08
**E-A48F7**	0.2±0.06	0.54±0.06	0.2±0.03	0	0.11±0.16	0.4±0.14
**E-A48F9**	1.36±0.05	0.13±0.04	0	0.5±0.1	0	1.0±0.04
**E-A48F10**	0.05±0.0	0	0.1±0.07	0.04±0.02	0	0.9±0.11
**E-A48F12**	0.3±0.03	0.24±0.1	0.1±0.01	0.11±0.01	0	0.13±0.01
**E-A50F1**	0	0	0	0	0	0
**F-A53F2**	0	0	0*	0.09±0.01	0.07±0.14	1.3±04
**H-A56F4**	0	0	0*	0	0	0.24±0.02
**H-A60F2**	0	0	0*	0.06±0.03	0.1±0.04	0.4±0.1
**F-A62F1**	0.8±0.08	0.5±0.03	0*	0.09±0.0	0.11±0.02	0.07±0.02
**F-A63F3**	3.63±0.08	0	0	0	0	0.33±0.0
**G-A68F2**	0.16±0.0	0.6±0.3	0	1.6±0.3	0	0.19±0.05
**G-A71F3**	0.6±0.15	0.84±0.07	0	1.2±0.11	0*	0.63±0.14
**G-A72F3**	0	0.23±0.03	0*	0	0	0.2±0.04

Data shown are representative of two independent experiments performed in four replicated (means ± standard deviation) with similar results from the two experiments
